# A new search for thermotolerant yeasts, its characterization and optimization using response surface methodology for ethanol production

**DOI:** 10.3389/fmicb.2015.00889

**Published:** 2015-09-01

**Authors:** Richa Arora, Shuvashish Behera, Nilesh K. Sharma, Sachin Kumar

**Affiliations:** ^1^Biochemical Conversion Division, Sardar Swaran Singh National Institute of Bio-EnergyKapurthala, India; ^2^I.K Gujral Punjab Technical UniversityKapurthala, India

**Keywords:** thermotolerant yeast, optimization, face-centered central composite design, bioethanol production, glucose, xylose

## Abstract

The progressive rise in energy crisis followed by green house gas (GHG) emissions is serving as the driving force for bioethanol production from renewable resources. Current bioethanol research focuses on lignocellulosic feedstocks as these are abundantly available, renewable, sustainable and exhibit no competition between the crops for food and fuel. However, the technologies in use have some drawbacks including incapability of pentose fermentation, reduced tolerance to products formed, costly processes, etc. Therefore, the present study was carried out with the objective of isolating hexose and pentose fermenting thermophilic/thermotolerant ethanologens with acceptable product yield. Two thermotolerant isolates, NIRE-K1 and NIRE-K3 were screened for fermenting both glucose and xylose and identified as *Kluyveromyces marxianus* NIRE-K1 and *K. marxianus* NIRE-K3. After optimization using Face-centered Central Composite Design (FCCD), the growth parameters like temperature and pH were found to be 45.17°C and 5.49, respectively for *K. marxianus* NIRE-K1 and 45.41°C and 5.24, respectively for *K. marxianus* NIRE-K3. Further, batch fermentations were carried out under optimized conditions, where *K. marxianus* NIRE-K3 was found to be superior over *K. marxianus* NIRE-K1. Ethanol yield (*Y*_*x*∕*s*_), sugar to ethanol conversion rate (%), microbial biomass concentration (*X*) and volumetric product productivity (*Q*_*p*_) obtained by *K. marxianus* NIRE-K3 were found to be 9.3, 9.55, 14.63, and 31.94% higher than that of *K. marxianus* NIRE-K1, respectively. This study revealed the promising potential of both the screened thermotolerant isolates for bioethanol production.

## Introduction

Production of ethanol is an important task worldwide for improving global energy demand. Bioethanol has been produced from different renewable feedstocks like fermentable sugars, starchy and lignocellulosic materials such as agricultural residues (paddy straw, wheat straw, sugarcane bagasse), woody plants and waste papers (Viikari et al., 2012; Behera et al., 2014a,b; Kumar et al., 2015). Currently, agricultural biomass is of the prime focus in the renewable energy production. The major components like cellulose and hemicellulose in lignocellulosic biomass can be converted to fermentable sugars such as glucose and xylose through saccharification, which can further be fermented to ethanol by various microorganisms (Kumar et al., 2009a; Pakarinen et al., 2011; Narayanaswamy et al., 2013).

Glucose is the most abundant sugar in lignocelluloses, which can be easily metabolized for the production of bioethanol. The yeast *Saccharomyces cerevisiae* is a traditional glucose fermenting yeast with the highest ethanol yield and tolerance (Behera and Ray, 2012). The second most abundant sugar is xylose which cannot be easily fermented by the industrial strains and can add up to 25% of the composition of lignocellulosic feedstocks (Olofsson et al., 2008). Several xylose fermenting yeasts such as *Pichia segobiensis, Scheffersomyces stipitis, Pachysolen tannophilus, Scheffersomyces shehatae, C. lyxosophila, C. prachuapensis, C. intermedia, C. tenuis C. jeffriesii, Brettanomyces naardenensis, Spathaspora passalidarum, Spathaspora arborariae*, etc. has been reported by different researchers (Cadete et al., 2009; Nitiyon et al., 2011; Lorliam et al., 2013). But the rate of xylose consumption is reported to be very low by these strains. However, utilization of both sugars present in lignocellulosic biomass is an important parameter to increase the production of ethanol. Co-fermentation of glucose and xylose sugars through co-cultivation of microbial strains shows unsatisfactory results due to their difference in ethanol tolerance and fermentation conditions (Jeffries, 2006).

Currently, majority of bioethanol is produced using mesophilic microorganisms. However, thermo-ethanologenic yeasts receive considerable interest due to current challenges of increasing temperature, which could potentially overcome many obstacles. The use of thermophillic/thermotolerant yeast for bioethanol production have several process advantages including broad substrate utilization range, higher saccharification and fermentation rates, minimized contamination risk, lower costs of pumping and stirring and no cooling problems, less energy requirement for mixing and product recovery (Dung et al., 2012; Kumar et al., 2013; Arora et al., 2015a; Scully and Orlygsson, 2015). The thermophilic/thermotolerant microorganisms produce distinctive enzymes that function under extreme conditions comparable to those existing in several industrial processes (Arora et al., 2015b). Hence, these microorganisms are of great interest for industrial applications.

Many researchers have reported isolation of thermoethanologenic species including *Kluyveromyces marxianus, Issatachenkia orientalis, Geobacillus thermoglucosidasius*, Clostridium sp., Thermoanaerobacterium AK54, Thermoanaerobacter *pentosaceus* (Riyanti and Rogers, 2009; Kwon et al., 2011; Orlygsson, 2012; Sigurbjornsdottir and Orlygsson, 2012; Tomás et al., 2013; Arora et al., 2014). Although, these species exhibit ethanol production at high temperature but still there are certain challenges including lower yield from pentoses, lower tolerance to ethanol, sensitivity to fermentation inhibitors and production of various by-products. Hence, optimization of growth and fermentation parameters is essential for higher fermentation efficiency.

Response Surface Methodology (RSM) is a statistical tool, which is used to design the experiments, build models, thereby, evaluate the effect of various variables on one or more responses and sets an optimal solution for the responses with reduction in the number of experimental runs (Bas and Boyaci, 2007; Uncu and Cekmecelioglu, 2011). Most commonly used design in RSM is Central Composite Design (CCD) which is characterized by flexible rotation in the design space, more precise predictions about the response of the variables along with the information about the experimental errors (Montgomery, 2009). Optimization using CCDs have been applied by many researchers for the optimization of various parameters for bioethanol production (Manikandan and Viruthagiri, 2010; Zhao et al., 2010; El-Gendy et al., 2013; Manivannan and Narendhirakannan, 2014).

The present study was carried out for the new search of thermotolerant/thermophilic co-fermenting yeasts for bioethanol production. The parameters like temperature and pH were optimized for growth and fermentation of isolated thermotolerant ethanologens with RSM using statistical software for enhancing their growth rate which results in augmented production of ethanol.

## Materials and methods

### Sample collection

Soil samples were collected from various sites in the different states of India (Table 1) by exploring each site for 60–90 min by walking-through in varying climatic conditions. Soil samples were placed in the plastic bags and their details were recorded. All the samples were stored in the refrigerator at 4 ± 0.5°C for further isolation process.

**Table 1:** **Microorganisms isolated from various sites for bioethanol production**.

**Sample collection site**	**Date and time of collection**	**Total isolates**	**Ethanologen positive in glucose**	**Ethanologen positive in xylose**
Manure soil from Ibban village, Kapurthala (31.3800°N, 75.3800°E)	9th Jul, 201212:00 noon–2:00 pm	11	–	–
Sugarcane juice sample from local retailer, Kapurthala (31.3800°N, 75.3800°E)	14th Jul, 201210:00 am–12:00 pm	14	–	–
Mahua slurry from pilot plant, Odisha (20.1500°N, 85.5000°E)	23rd Aug, 201211:00 am–2:30 pm	7	2	–
Soil samples from different sites in a sugarmill in Phagwara (31.2200°N, 75.7700°E)	10th Sep, 201311:00 am–12:30 pm	5	2	–
Soil samples from different sites in a sugarmill in Karnal (26.6900°N, 76.9800°E)	16th Sep, 201310:00 am–11:30 am	7	2	2
Kitchen waste samples, NIRE campus, Kapurthala (31.3800°N, 75.3800°E)	16th Oct, 201310:30 am-11:30 am	28	4	–
Soil samples from decaying wood in different areas in Jalandhar (31.3260°N, 75.5760°E)	20th Oct, 201310:00 am–1:00 pm	10	–	–
Rotten fruits from NIRE campus, Kapurthala (31.3800°N, 75.3800°E)	22nd Jun, 20149:00 am–9:30 am	5	–	–
Insects from tree trunks, Kapurthala (31.3800°N, 75.3800°E)	24th Oct, 20149:00 am–10:00 am	4	1	–
Dumpyard, Kapurthala (31.3800°N, 75.3800°E)	28th Oct, 20141:00 pm–2:00 pm	12	3	-

### Isolation, screening and identification of yeasts for ethanol production

A pinch of soil sample was suspended in 10 ml of sterile water and vortexed for 2 min at maximum speed before 10 × serial dilution. 100 μl from each dilution in series was spread onto the surface of yeast extract-peptone (YEP) phytagel (dextrose, 2%; yeast extract, 1%; peptone, 2%; phytagel, 1.5%; ampicillin, 50 mg/ml; pH, 5.5) plates having 1% glucose or xylose sugar and incubated at different temperature (40–50°C) for 24–48 h. Xylose utilizing yeasts were isolated by using xylose sugar (2%) in place of dextrose to the above medium. Based on the morphology, size and color, various colonies were selected and subcultured on to separate phytagel plates to ensure their purity. The isolated strains were carefully studied on the basis of cultural characteristics the colony and their growth pattern. Strain selection was based on the anaerobic growth and production of ethanol on media containing both glucose as well as xylose. Further, the selective yeast cultures were identified on the basis of sequencing of Internal transcribed spacer (ITS) and D1D2 domain of rRNA gene from Microbial Type Culture Collection and Gene Bank (MTCC), Chandigarh, India. The homologies of the sequences were determined using the Basic Local Alignment Search Tool (BLAST) system of the DNA Data Bank of National Center for Biotechnology Information (NCBI).

### Microorganism and culture condition

The isolated yeast cultures were maintained on YEP medium having dextrose (YEPD) or xylose (YEPX) sugar according to the use by the isolates. The culture was stored at 4 ± 0.5°C for further use. The stock culture was maintained in glycerol 30% at −80°C.

### Growth conditions

The inoculums for both *K. marxianus* NIRE-K1 and NIRE-K3 were prepared in 100 ml salt medium with composition (in g l^−1^) ammonium sulfate, 2.0; di-sodium hydrogen ortho phosphate, 0.15; potassium di-hydrogen ortho phosphate, 0.15; yeast extract, 1.0; glucose, 10.0 at pH 5.5, 45°C, taken in sterilized (at 121°C for 20 min) 500 mL erlenmeyer flask. The flask was inoculated with 100 μl of mother inoculums from YP medium and incubated for 24 h at 150 rpm in an orbital shaker incubator. The cells grown after 24 h were used as the inoculum used for subsequent runs for optimization.

### Optimization of temperature and pH using FCCD

Physical and chemical variables essential for growth of both the screened isolates *K. marxianus* NIRE-K1 and NIRE-K3 were optimized according to RSM using Design Expert software version 8.0 (STAT-EASE Inc., Minneapolis, USA). FCCD was employed to study the combined effect of temperature and pH on the maximum specific growth rate (μ_max_) as the response. Both the growth parameters were studied at three levels viz. low (−1), middle (0) and high (+1), with alpha value of 1. The real and coded values of these variables applicable for both the isolates have been presented in Table 2. The software was designed with 13 experimental runs with 5 runs at the center points to ensure the reproducibility of the model. The responses, μ_max_ for both the cultures, were calculated using Monod model from the Equation (1), as described below:

(1) $$\begin{array}{l} \mu =\;\frac{\mu_{\max}S}{K_{s}+S}\; \end{array}$$

**Table 2:** **Coded values for each variable of FCCD for growth**.

**Variables**	**Unit**	**−1**	**0**	**+1**
Temperature	°C	37	43.5	50
pH	–	3.5	5.5	7.5

Where, μ is the specific growth rate (h^−1^), *S* is rate limiting substrate concentration (g l^−1^), *K*_*s*_ is saturation constant or half velocity constant or substrate utilization constant (g l^−1^). The values of the responses were the means of two replications to enhance the accuracy of the model.

After regression analysis, the statistical significance of the model was evaluated using analysis of variance (ANOVA) and lack of fit tests. The variables that significantly affected the responses were determined using a confidence level above 95% or a *p*-value less than 0.05. The response of the dependent variable was evaluated using the second order polynomial Equation (2) with variance for each variable divided into linear, quadratic and interactive components as described below:

(2) $$\begin{array}{l} Y=b_{0}+b_{1}x_{1}+b_{2}x_{2}+b_{11}{x_{1}}^{2}+b_{22}{x_{2}}^{2}+b_{12}x_{1}x_{2}\; \end{array}$$

Where, *Y* is predicted response (μ_max_, h^−1^), *x*_1_ and *x*_2_ are the coded levels of independent variables, *b*_0_ is the offset term, *b*_1_ and *b*_2_ are the linear effects, *b*_11_ and *b*_22_ are the quadratic effects and *b*_12_ is the interaction effect.

The quality of the model developed was estimated by coefficient of determination (*R*^2^), adjusted *R*^2^ (*R*${}_{\text{adj}}^{2}$) and predicted *R*^2^ (*R*${}_{\text{predict}}^{2}$). Contour and three dimensional plots were drawn to illustrate the relationship between the responses and the variables to be optimized. The interaction of one parameter with other can be studies from the pattern of the contour plots. For obtaining the optimal solutions, numerical optimization method of Design Expert software was employed.

### Model validity

To validate the authenticity of software generated model, a confirmatory experiment with duplicate sets was performed under the optimized conditions to verify the predicted values for maximum specific growth rate.

### Fermentation conditions

Inoculums for fermentation by isolates *K. marxianus* NIRE-K1 and *K. marxianus* NIRE-K3 were prepared in the salt medium with similar composition as that of growth medium containing 10 g l^−1^ glucose concentration. The cells from the exponential phase were pumped into a bioreactor of 5 L working volume (NBS BioFlo-CelliGen 115) with initial glucose concentration of 100 g l^−1^. The pH and temperature for both the isolates were maintained at optimized conditions suggested by the software.

### Analytical procedures

Samples were withdrawn at 4 h intervals and centrifuged at 10,000 rpm for 15 min. The collected samples were stored at 4°C prior to analysis. Various metabolites in fermentation broth (glucose, ethanol, glycerol, and acetic acid) were analyzed using High Performance Liquid Chromatography (Agilent Technologies) using HiPlex H column at 57°C with 1 mM H_2_SO_4_ as the mobile phase at a flow rate of 0.7 ml min^−1^ and detected by Refractive Index Detector at 50°C.

Dry cell weight (DCW) was obtained by centrifuging 1 ml of sample in pre-dry weighted Eppendorf tube using Eppendorf centrifuge 5430 R at 10,000 rpm for 15 min, followed by washing the pellet twice with distilled water and further drying in a vacuum oven at 80°C to a constant weight. All the experiments were carried out in triplicate, and the given values are the mean values. Fermentation kinetics for both the screened isolates was calculated using the formulae by Bailey and Ollis (1986).

## Results and discussion

### Isolation, screening and identification of yeasts for ethanol production

A wide variety of microorganisms are present in the environment, which can be harnessed for the purpose of mankind. The isolation and screening of the efficient therrmophilic/thermotolerant ethanol producing yeasts can be helpful to overcome the current challenges in biofuel production. Therefore, the thermophilic/thermotolerant character was taken into the consideration in this study. A total of 103 thermophilic strains of yeast were isolated from different samples collected from various sites (Table 1). The isolated strains were carefully identified by cultural characteristics and growth pattern studies using microscope. About 14 yeast isolates were found to be positive for ethanol production from glucose. However, in fermentation studies on defined media for screening of isolates, two yeasts (NIRE-K1 and NIRE-K3) showed to possess the ability to ferment both glucose and xylose to ethanol under anaerobic conditions (Table 1).

**Figure 1:**
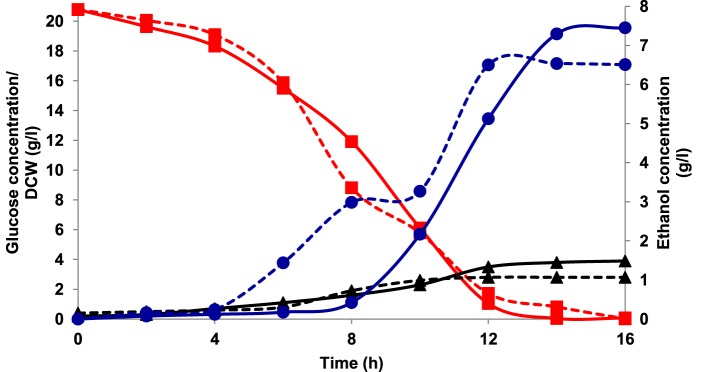
**Growth of *K. marxianus* NIRE-K1 and NIRE-K3 in YEPD medium, (---) *K. marxianus* NIRE-K1; (—) *K. marxianus* NIRE-K3; (■) Glucose; (▴) Dry cell weight (DCW); (•) Ethanol**.

**Figure 2:**
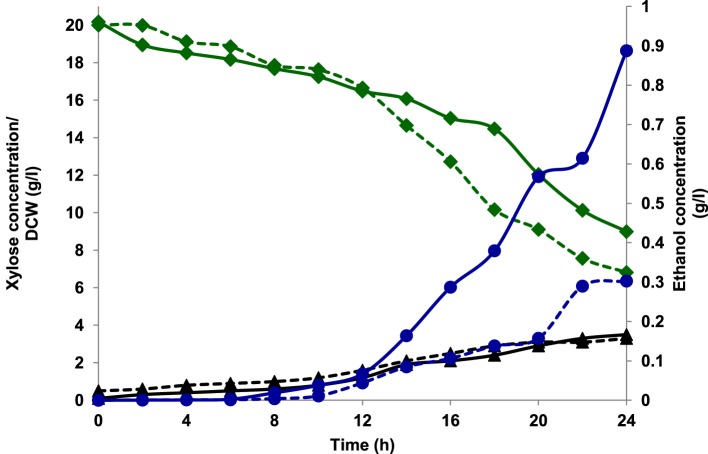
**Growth of *K. marxianus* NIRE-K1 and NIRE-K3 in YEPX medium, (---) *K. marxianus* NIRE-K1; (—) *K. marxianus* NIRE-K3; (♦) Xylose; (▴) Dry cell weight (DCW); (•) Ethanol**.

**Figure 3:**
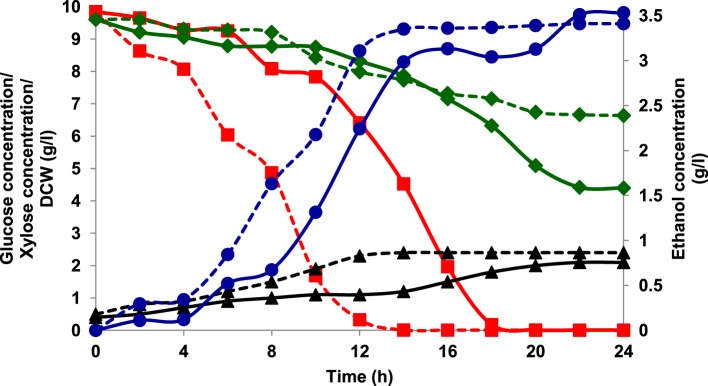
**Growth of *K. marxianus* NIRE-K1 and NIRE-K3 in YEPDX medium, (---) *K. marxianus* NIRE-K1; (—) *K. marxianus* NIRE-K3; (■) Glucose; (♦) Xylose; (▴) Dry cell weight (DCW); (•) Ethanol**.

Aerobic batch fermentations were carried out using both the yeast strains of *K. marxianus* NIRE-K1 and NIRE-K3 on YEP medium containing 2% glucose and xylose solely and further mixture of both in equal ratio at 45°C. Both the strains of *K. marxianus* NIRE-K1 and NIRE-K3 could produce maximum ethanol concentration with ethanol yield of 0.31 ± 0.023 and 0.36 ± 0.022 g g^−1^, respectively in YEPD medium with complete utilization of glucose (20 g l^−1^) in 16 h (Figure 1). However, both the strains could utilize xylose for the production of ethanol with concomitant xylitol production. In case of xylose containing YEPX media using cells of *K. marxianus* NIRE-K1, maximum ethanol and xylitol concentration of 0.3 ± 0.01 and 4.34 ± 0.03 g l^−1^ were obtained in 24 h of duration through the utilization of 13.18 ± 0.029 g l^−1^ xylose sugar (Figure 2). Similarly with the same duration of fermentation, *K. marxianus* NIRE-K3 could be able to utilize 11.18 ± 0.04 g l^−1^ of xylose for the production of 0.88 ± 0.01 and 0.80 ± 0.01 g l^−1^ of ethanol and xylitol, respectively (Figure 2). In other hand, both the strains of *K. marxianus* NIRE-K1 and NIRE-K3 was capable of simultaneously using mixture of glucose and xylose in YEPDX medium, achieving maximum ethanol concentration of 3.4 ± 0.051 and 3.5 ± 0.057 g l^−1^, respectively in 24 h of fermentation. Both the strains could be able to completely utilize glucose, while 6.63 ± 0.1 and 4.39 ± 0.01 g l^−1^ of residual xylose was left in case of *K. marxianus* NIRE-K1 and NIRE-K3, respectively (Figure 3).

On the basis of above study, these two isolates were selected, identified and optimized for further study. Both the screened isolates, NIRE-K1 and NIRE-K3 were sequenced and identified as *Kluyveromyces marxianus* NIRE-K1 and *Kluyveromyces marxianus* NIRE-K3, respectively. Both the cultures, *K. marxianus* NIRE-K1 and *K. marxianus* NIRE-K3 have been deposited at MTCC, Chandigarh with the deposition no. MTCC 5933 and MTCC 5934, respectively. Subsequently, partial genome sequence of *Kluyveromyces marxianus* NIRE-K1 and NIRE-K3 (ascomycetes yeasts of the fungal family Saccharomycetaceae and order Saccharomycetales) have been submitted in NCBI gene bank with GenBank accession number **KP405925.1** and **KP405926.1**, respectively.

**Figure 4:**
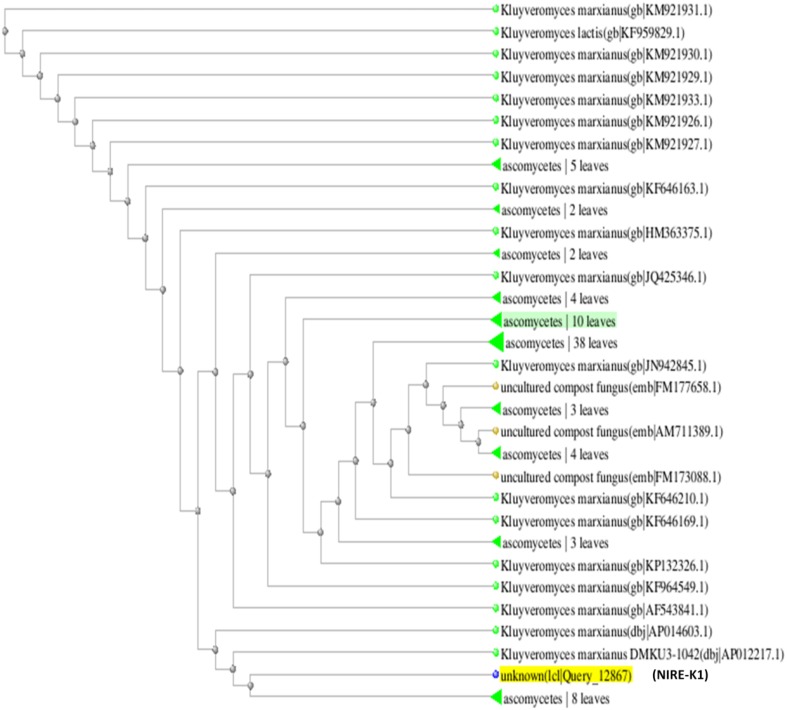
**Phylogenetic tree drawn through BLAST showing genetic relationship between *K. marxianus* NIRE-K1 and similar organisms**.

**Figure 5:**
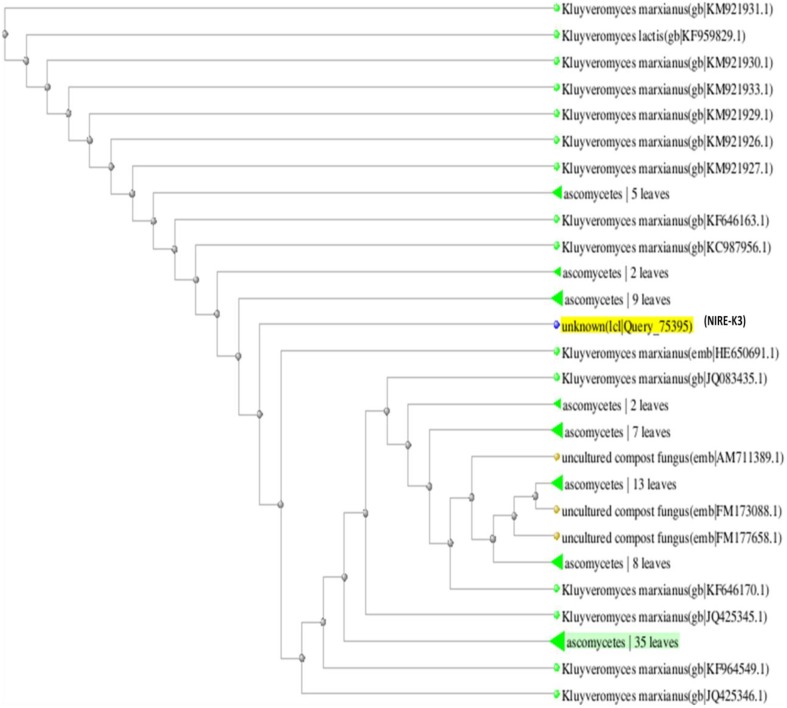
**Phylogenetic tree drawn through BLAST showing genetic relationship between *K. marxianus* NIRE-K3 and similar organisms**.

The phylogenetic tree is the study of evolutionary relatedness between the species. In this study, phylogenetic tree was drawn on the basis of distance matrix of homology sequence of similar microorganisms by BLAST where isolate NIRE-K1 was found to be related to the ascomycetes group and have maximum homology similarity with *K. marxianus* strain CHY1612 which has been depicted in Figure 4. Similarly, other isolate NIRE-K3 showed maximum similarity with both the strains of *K. marxianus* 1.2 18S and *K. marxianus* B.WHX.12 (Figure 5). Further, sequence of NIRE-K1 is the substem of *K. marxianus* DMKU3-1042 with 99% homology. Therefore, on the basis of the above similarity, the isolate NIRE-K1 and NIRE-K3 were confirmed as the *K. marxianus* which were named as *K. marxianus* NIRE-K1 and *K. marxianus* NIRE-K3.

Several researchers have isolated various yeasts from different sources for the ethanol production to reduce the overall cost. Yeast cells exhibit a complex mechanism with rapid molecular response, when exposed to elevated temperature (Cimpeanu et al., 2010; Ma and Liu, 2010; Stanley et al., 2010). Tofighi et al. (2014) isolated thermotolerant *Saccharomyces cerevisiae* from waste water samples with optimum growth temperatures ranging from 35 and 40°C and ethanol yield of 75% of its theoretical value in respect to glucose. Kaewkrajay et al. (2014) isolated thermotolerant yeast from the soil samples collected from sugarcane, cassava and pineapple plantations in five different provinces of Thailand. The yeast was used for ethanol production from cassava starch hydrolysate at 45°C with the maximum ethanol concentration of 42.4 g l^−1^ in 48 h, at a productivity of 0.88 g l^−1^ h^−1^ and a yield of 46% of the theoretical yield in respect to glucose. Similarly, Yuangsaard et al. (2013) isolated *Pichia kudriavzevii* DMKU 3-ET15 from the traditional fermented pork sausage, which produced ethanol concentration of 4% (w/v) with productivity of 1.27 g l^−1^ h^−1^ and yield of 42% of the theoretical yield in a cassava starch hydrolysate medium at pH 5.0 and 45°C. Dhaliwal et al. (2011) reported the isolation of *P. kudriavzevii* from sugarcane juice at 40°C, which produced 71.9 g/l of ethanol with a productivity of 4.0 g l^−1^ h^−1^. Hashem et al. (2013) isolated two thermotolerant yeasts *Kluyveromyces* sp. GU133329 and *Kluyveromyces* sp. GU133331 from plum fruit and cantaloupe, which produced 9.55 (w/v) and 11.72% (w/v) of ethanol, respectively at pH 5.5 and temperature of 35°C. These results imply that these isolated strains could be able to produce ethanol at high temperature.

### Optimization of growth conditions

Physical and chemical parameters viz. temperature and pH play a noteworthy role in controlling the growth of microorganisms (Charoenchai et al., 1998). Growth and fermentation rate increases to a certain extent with increase of both temperature and pH, which decreases sharply, thereby lowering both cell and ethanol yields (Torija et al., 2003; Phisalaphong et al., 2005). According to Anusiem (2004), with every 10°C rise in temperature, the rate of reaction is doubled. However, in case of biochemical reactions, the inhibition of temperature on growth and fermentation beyond the optimized range can be endorsed due to denaturation of ribosomes and the enzymes as well as disruption of the cell membrane due to changes in the fluidity (McMeckin et al., 2002). Similarly, the deleterious effect of pH beyond the optimized range could influence the NADH to NAD^+^ ratio, which further affects the metabolic flux toward ethanol and biomass formation (Peña et al., 1972; Adnan et al., 2014). Hence, both these factors must be studied with respect to their interactive behavior and their influence on biomass and ethanol yields.

In the present study, both the variables were taken into account to study their effect on the specific growth rate of both the screened yeast isolates. FCCD matrix for both the variables (temperature and pH) with experimental and predicted values of maximum specific growth rates of the isolates *K. marxianus* NIRE-K1 and NIRE-K3 has been shown in Table 3. The significance of the quadratic model was further determined by the ANOVA tables through Fisher's “*F*”-test (Tables 4, 5). The regression models (quadratic) were found to be significant with *F*-value of 305.00 and 244.04 for *K. marxianus* NIRE-K1 and NIRE-K3, respectively. Moreover, for both the isolates, *p*-values were found to be less than 0.05. According to Rene et al. (2007), the high significance of the regression model is indicated by the *F*-value with a low probability “*p*”-value. For both the isolates, linear effect of temperature and squared effect of both temperature and pH were found to be significant model terms with *p* < 0.05 (Tables 4, 5). Though the variable, pH is insignificant in linear term but its significance in the squared term shows that any change in these variables significantly affects the specific growth rate. On the contrary, Serra et al. (2005) specified temperature as the main influencing factor in comparison to pH on maximum specific growth rate of wine yeasts, *S. bayanus* var. *uvarum* P3 and *S. cerevisiae* VL3c. The effect of both temperature and pH for ethanol production is also reported by Eiadpum et al. (2012), Singh and Bishnoi (2012), and Udhayaraja and Sriman (2012).

**Table 3:** **Experimental data and results of FCCD for the growth of *K. marxianus* NIRE-K1 and NIRE-K3**.

**Run**	**Temperature (°C)**	**pH**	**μ_max_ for *K. marxianus* NIRE-K1 (h^−1^)**		**μ_max_ for *K. marxianus* NIRE-K3 (h^−1^)**	
			**Experimental value**	**Predicted value**	**Experimental value**	**Predicted value**
1	37.00	3.50	0.400	0.395	0.140	0.140
2	50.00	3.50	0.000	−0.016	0.000	−0.007
3	37.00	7.50	0.380	0.380	0.110	0.110
4	50.00	7.50	0.000	−0.008	0.000	−0.009
5	37.00	5.50	0.420	0.420	0.160	0.157
6	50.00	5.50	0.000	0.024	0.000	0.016
7	43.50	3.50	0.440	0.460	0.230	0.230
8	43.50	7.50	0.450	0.460	0.210	0.220
9	43.50	5.50	0.490	0.500	0.250	0.250
10	43.50	5.50	0.520	0.500	0.240	0.250
11	43.50	5.50	0.500	0.500	0.260	0.250
12	43.50	5.50	0.510	0.500	0.247	0.250
13	43.50	5.50	0.490	0.500	0.251	0.250

**Table 4:** **ANOVA for the experimental results of the FCCD for *K. marxianus* NIRE-K1**.

**Source**	**Sum of Squares**	**df^a^**	**Mean square**	***F*-value**	***P*-value Prob >*F***	
Model	0.51	5	0.10	305.00	< 0.0001	Significant
A-Temperature	0.24	1	0.24	721.07	< 0.0001	
B-pH	0.00001667	1	0.00001667	0.050	0.8293	
AB	0.0001	1	0.0001	0.30	0.6006	
A^2^	0.20	1	0.20	612.67	< 0.0001	
B^2^	0.003725	1	0.003725	11.19	0.0123	
Residual	0.00233	7	0.0003328			
Lack of Fit	0.00165	3	0.0005500	3.24	0.1432	Not significant
Pure error	0.00068	4	0.00017			
Cor Total	0.51	12				

a df, Degrees of freedom; F, Fisher's variance ratio; P, probability value; Cor Total, Totals corrected for the mean; P < 0.05- significant at 5% level; R^2^ = 0.9954; Adjusted R^2^ = 0,9922; Predicted R^2^ = 0.9731; Adequate precision = 41.292; PRESS = 0.014; CV = 5.16%.

**Table 5:** **ANOVA for the experimental results of the FCCD for *K. marxianus* NIRE-K3**.

**Source**	**Sum of Squares**	**df^a^**	**Mean square**	***F*-value**	***P*-value Prob >*F***	
Model	0.13	5	0.025	244.04	< 0.0001	Significant
A-Temperature	0.028	1	0.028	269.82	< 0.0001	
B-pH	0.0004167	1	0.0004167	4.01	0.0852	
AB	0.000225	1	0.000225	2.17	0.1845	
A^2^	0.074	1	0.074	713.31	< 0.0001	
B^2^	0.001559	1	0.001559	15.01	0.0061	
Residual	0.0007268	7	0.0001038			
Lack of Fit	0.0005176	3	0.0001725	3.30	0.1395	Not significant
Pure error	0.0002092	4	0.0000523			
Cor Total	0.13	12				

a df, Degrees of freedom; F, Fisher's variance ratio; P, probability value; Cor Total, Totals corrected for the mean; P < 0.05–significant at 5% level; R^2^ = 0.9943; Adjusted R^2^ = 0.9902; Predicted R^2^ = 0.9589; Adequate precision = 37.079; PRESS = 0.005236; CV = 6.31%.

The “lack of fit” was found to be non-significant relative to the pure error with *F*-value of 3.24 and 3.30, respectively for both the isolates, *K. marxianus* NIRE-K1 and NIRE-K3. The equations based on the quadratic models for the isolates, *K. marxianus* NIRE-K1 (Equation 3) and NIRE-K3 (Equation 4), respectively, in terms of experimental factors has been represented below:

(3) $$\begin{array}{rcl} Y & = & -10.52+0.53\times A+0.08\times B+3.85\times E-004\times A\times B\\ -6.43\times E-003\times A^{2}-9.18\times E-003\times B^{2} \end{array}$$

(4) $$\begin{array}{rcl} Y & = & -6.65+0.32\times A+0.04\times B+5.77\times E-004\times A\times B\\ -3.88\times E-003\times A^{2}-5.94\times E-003\times B^{2} \end{array}$$

Where, *Y* is the maximum specific growth rate (h^−1^); *A* and *B* are temperature and pH, respectively.

The goodness of fit of the regression model is estimated by the coefficient of determination (*R*^2^) which measures the variability in the response values due to variation in the experimental factors and their interactions. The R-squared values close to 1 indicates the stronger model and better response prediction (Ohtani, 2000). However, a model can be accepted with *R*^2^ > 0.75 (Chauhan and Gupta, 2004). The model presented in Table 4 for *K. marxianus* NIRE-K1 exhibits high R-squared value of 0.9954 which explains 99.54% of the variation in the response, as well as high value of the adjusted determination coefficient (adjusted *R*^2^ = 0.9922) showing correlation between the observed and predicted values, suggesting a high significance of the model. Similar results were obtained for *K. marxianus* NIRE-K3 with determination coefficient (*R*^2^ = 0.9943) and adjusted determination coefficient (*R*${}_{\text{adj}}^{2}=0.9902$) as shown in Table 5. Singh et al. (2011) determined the accuracy of the RSM model by evaluating the correlation between the observed and predicted values, which was found to be ~0.9. However, the task of comparing the results of models from literature is quite exigent due to variation in operating conditions viz, type of inoculums, substrate, supplementary nutrients, type of reactor and its size.

**Figure 6:**
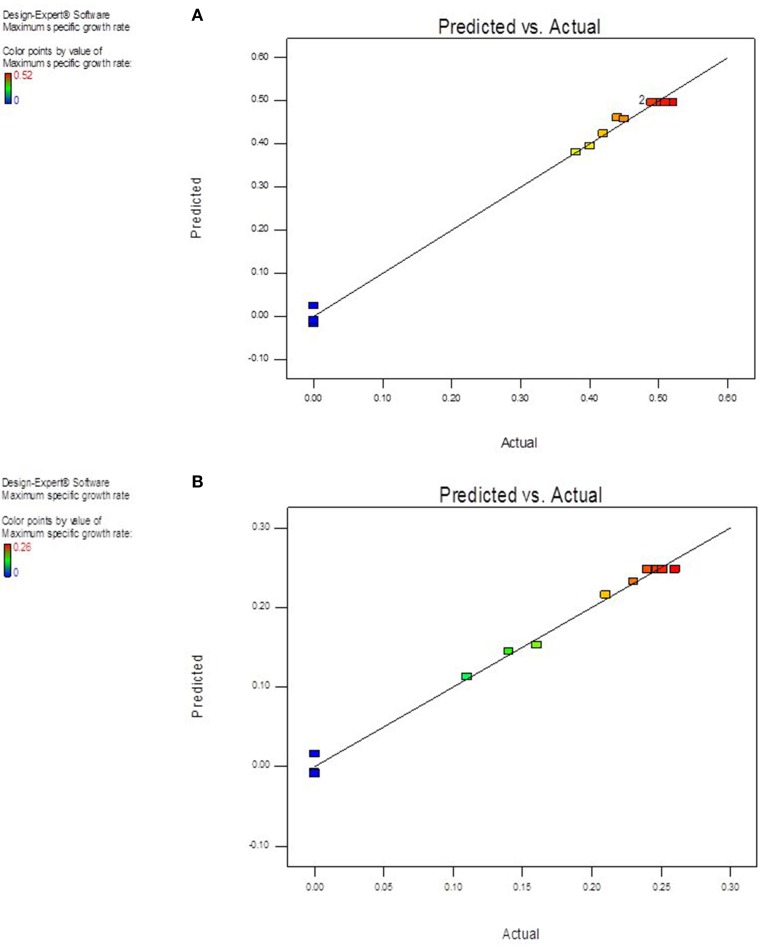
**Diagnostic plot of the distribution of observed and predicted values of maximum specific growth rate (A) *K. marxianus* NIRE-K1 (B) *K. marxianus* NIRE-K3**.

**Figure 7:**
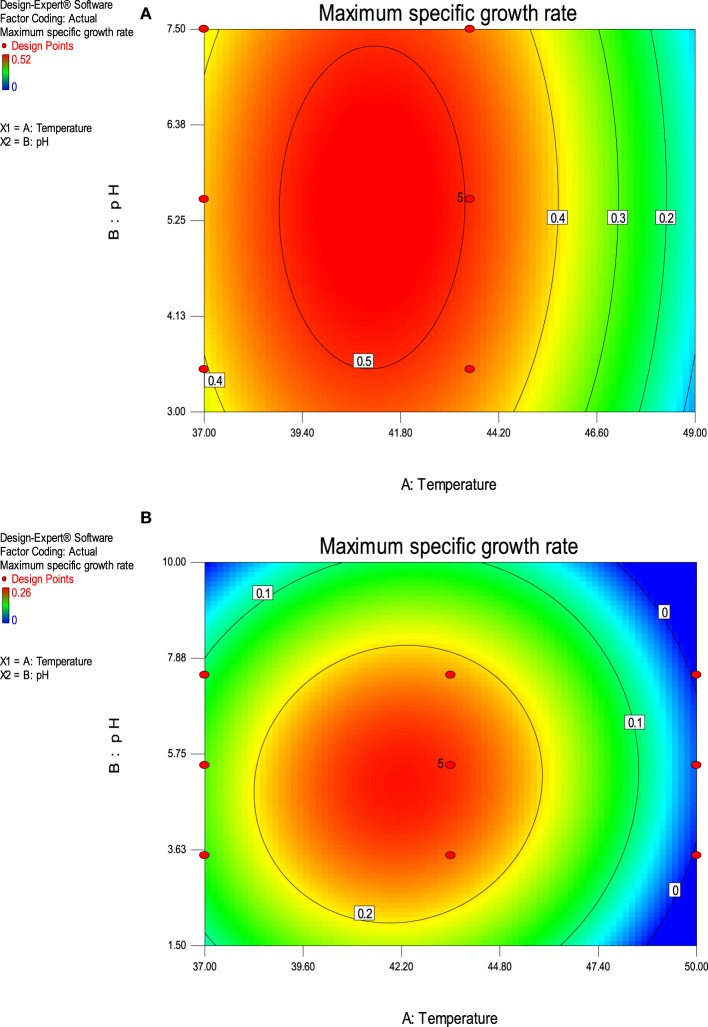
**Contour plot of maximum specific growth rate as a function of temperature and pH (A) *K. marxianus* NIRE-K1 (B) *K. marxianus* NIRE-K3**.

**Figure 8:**
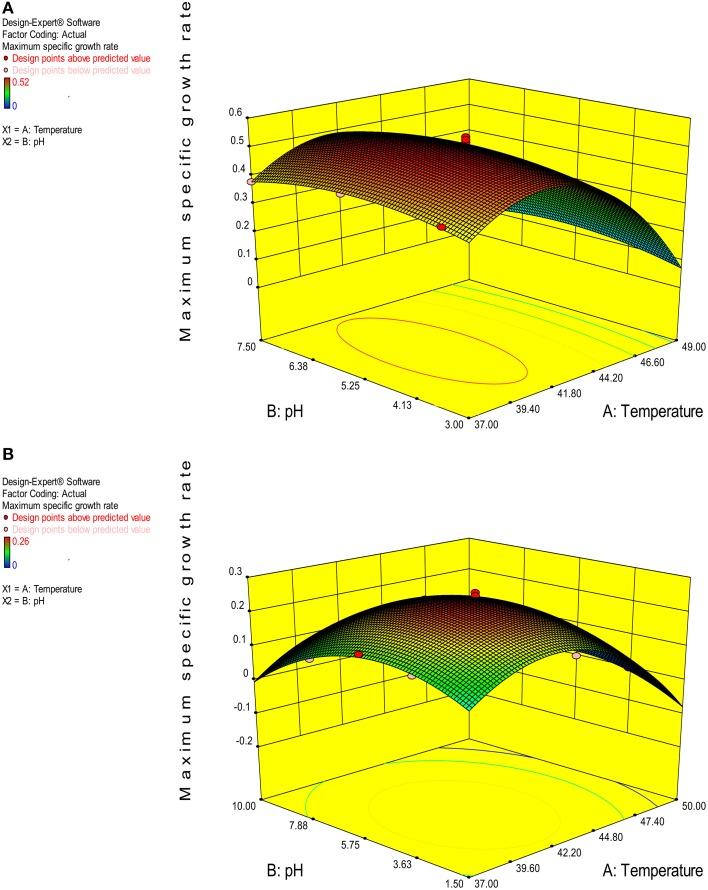
**3-D plot of maximum specific growth rate as a function of temperature and pH (A) *K. marxianus* NIRE-K1 (B) *K. marxianus* NIRE-K3**.

The value of coefficient of variation (*CV* = 5.16 and 6.31% for *K. marxianus* NIRE-K1 and NIRE-K3) was low due to the small residue between actual and predicted maximum specific growth rate. Also, to measure the adequate precision of the model and reliability of the experimental part, ratio of signal to noise is determined, where a ratio greater than 4 is desirable (Montgomery, 2001). In the present study, the ratio of 41.292 and 37.079 in case of *K. marxianus* NIRE-K1 and NIRE-K3 indicates an adequate signal to use the model for prediction purposes. Figures 6A,B show the diagnostic plots between the experimental and predicted values for *K. marxianus* NIRE-K1 and NIRE-K3, respectively, wherein all the points lie along the diagonal line, again indicating a good fit for both the models. Contour and three dimensional plots for isolates *K. marxianus* NIRE-K1 and NIRE-K3 have been shown in Figures 7, 8, respectively. The interaction between the variables is indicated by the shape of the contour plot. Strong interactions between the variables are indicated by the elliptical plots whereas circular plots indicate weaker interactions (Prakash et al., 2008). However, the plots of both the isolates were found to be elliptical (Figure 7). In three dimensional plots, (Figure 8), the convex response surface suggested well-defined optimum variables (temperature and pH) and maximum specific growth rate increased to the peak with the increase of temperature and pH up to 45°C and 5.5 for both the isolates which declined beyond the values.

The optimized values for both the variables were obtained by numerical optimization in the software with a desirability function values of 0.718 and 0.730 for *K. marxianus* NIRE-K1 and NIRE-K3, respectively. The optimized values of pH and temperature were found to be 5.49 and 45.17°C with a predicted μ_max_ of 0.427 in case of isolate *K. marxianus* NIRE-K1 which was 5.24 and 45.41°C for *K. marxianus* NIRE-K3 with a predicted μ_max_ of 0.214, keeping the goal of maximum temperature and specific growth rate. Similarly, Arroyo-López et al. (2009) reported 0.551 and 0.660 h^−1^ of maximum specific growth rates for yeasts T73 and hybrid W27 using optimized parameters of temperature 34.1°C and pH 4.76, respecively. Apart from the growth rate, many researchers have optimized the physical and chemical variables for ethanol fermentation using RSM. Man et al. (2010) reported maximum ethanol concentration of 24.17 g/L after optimizing the temperature of 38°C and pH 5.45. Dasgupta et al. (2013) optimized and reported a pH of 4.5 for *K. marxianus* IIPE453 to get maximum ethanol concentration.

### Validation of the model

To verify the accuracy of the model and reproduce the results predicted by the software, verification experiments were performed under optimized conditions for both the isolates and analyzed for their respective growth rates. The predicted growth rates suggested by the software under the optimized conditions were found to be 0.427 and 0.214 h^−1^ for *K. marxianus* NIRE-K1 and NIRE-K3, respectively. However, maximum specific growth rates of 0.413 and 0.209 h^−1^ were obtained after performing the experiments in triplicate for *K. marxianus* NIRE-K1 and NIRE-K3. The obtained values are in close agreement with the predicted values at a difference of only 3.3 and 2.3% for *K. marxianus* NIRE-K1 and NIRE-K3, respectively. According to Levin et al. (2008) differences between experimental and predicted values of less than 10% confirmed the validity of a model. Hence, the model developed from the response surface methodology in the present study is reliable and reproducible.

### Batch fermentation

**Figure 9:**
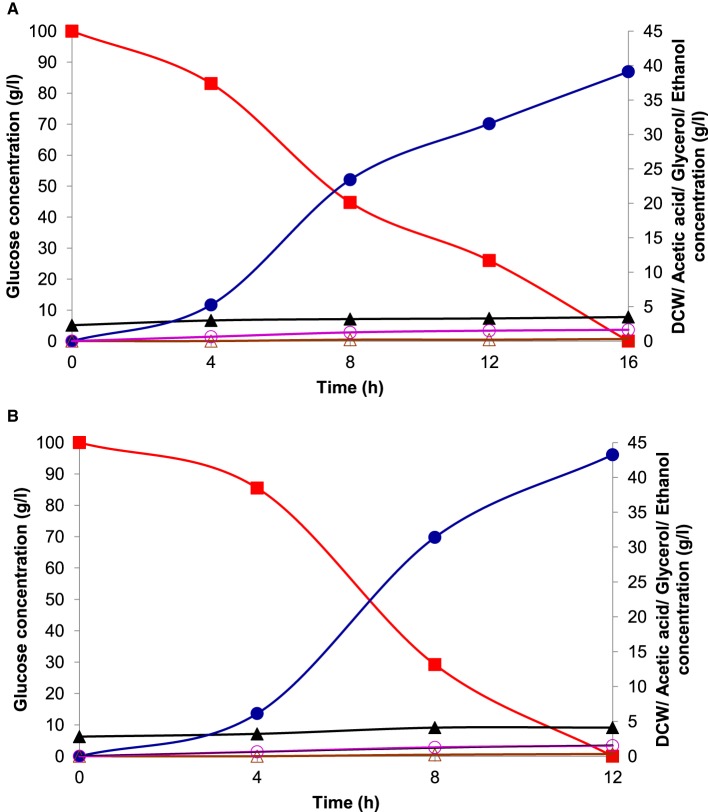
**Fermentation profile of (A) *K. marxianus* NIRE-K1 and (B) *K. marxianus* NIRE-K3 in bench-scale bioreactor at optimized growth conditions, (■) Glucose; (▴) Dry cell weight; (•) Ethanol; (○) Glycerol; (△) Acetic acid**.

Batch ethanol fermentation was performed under the optimized conditions of temperature and pH for fermentation with initial glucose concentration of 100 g l^−1^ by both the cultures of *K. marxianus* NIRE-K1 and *K. marxianus* NIRE-K3. The ethanol production and sugar utilization profile by these two cultures have been given in Figure 9. In the present study, ethanol production by both of the cells initiated in the log phage of the growth producing 5.26 ± 0.05 and 6.13 ± 0.18 g l^−1^ of ethanol using 16.89 ± 0.36 and 14.5 ± 0.33 g l^−1^ of sugar in 4 h of fermentation. The decrease in sugar concentration might be due to its utilization for initial growth and metabolism of the yeast in addition to its conversion into ethanol (Behera et al., 2011). For 8 and 12 h of fermentation, 55.26 and 74% of sugar was utilized with simultaneous increase in ethanol concentration to 23.44 ± 0.25 and 31.57 ± 0.48 g l^−1^, respectively with the cells of *K. marxianus* NIRE-K1 (Figure 9A). During 8 h of fermentation, 70.79% of sugar was utilized with simultaneous increase in ethanol concentration to 31.4 ± 0.40 g l^−1^ using the cells of *K. marxianus* NIRE-K3 (Figure 9B). Finally, maximum ethanol production of 39.12 ± 0.34 and 43.25 ± 0.36 g l^−1^ was achieved with 100% sugar utilization after 12 and 16 h of fermentation using the cells of *K. marxianus* NIRE-K1 and NIRE-K3, respectively.

The growth and fermentation kinetics of *K. marxianus* NIRE-K1 and NIRE-K3 were also studied which has been depicted in Table 6. The ethanol concentration (*P*) and volumetric substrate uptake (*Q*_*S*_) obtained with the cells of *K. marxianus* NIRE-K3 (43.25 ± 0.36 g l^−1^ and 8.33 ± 0.07 g l^−1^ h^−1^) was 9.6 and 24.97% more than that of *K. marxianus* NIRE-K1 cells (39.12 ± 0.34 g l^−1^ and 6.25 ± 0.028 g l^−1^ h^−1^). The ethanol yield (*Y*_*P*∕*S*_ = 0.43 ± 0.05 g g^−1^) and volumetric product productivity (*Q*_*P*_ = 3.6 ± 0.11 g l^−1^ h^−1^) obtained with *K. marxianus* NIRE-K3 cells was found to be 9.3 and 31.94%, respectively, higher than that of *Y*_*P*∕*S*_ (0.39 ± 0.12 g g^−1^) and *Q*_*P*_ (2.45 ± 0.17 g l^−1^ h^−1^) of *K. marxianus* NIRE-K1 cells. Likewise, the final biomass concentration (*X* = 4.1 g l^−1^) and sugar to ethanol conversion rate (86.5 ± 0.34%) with the *K. marxianus* NIRE-K3 cells was 14.63 and 9.55% more than that of *K. marxianus* NIRE-K1 cells. However, specific product formation rate (*q*_*p*_ = 0.798 ± 0.06 g g^−1^ h^−1^) and the specific sugar consumption rate (*q*_*s*_ = 1.85 ± 0.09 g g^−1^ h^−1^) in the case of *K. marxianus* NIRE-K3 cells was considerably 5.9 and 14.75% lower than the *K. marxianus* NIRE-K1 cells (*q*_*p*_ = 0.848 ± 0.05 g g^−1^ h^−1^ and *q*_*s*_ = 2.17 ± 0.19 g g^−1^ h^−1^), which is useful during product separation and purification process (Behera et al., 2010). The isolates *K. marxianus* NIRE-K1 and NIRE-K3 showed 5.13 and 9.3% higher ethanol yield with optimized parameters as compared to without optimized one (*Y*_*P*∕*S*_ = 0.37 ± 0.05 and 0.39 ± 0.07 g g^−1^ in case on *K. marxianus* NIRE-K1 and NIRE-K3).

**Table 6:** **Growth and fermentation kinetics of *K. marxianus* NIRE-K1 and NIRE-K3 at optimized conditions**.

	***K. marxianus* NIRE-K1**	***K. marxianus* NIRE-K3**
Initial sugar concentration (*S*, g l^−1^)	100.00 ± 0.03	100.00 ± 0.02
Final ethanol (*P*, g l^−1^)	39.12 ± 0.34	43.25 ± 0.36
Final biomass concentration (*X*, g l^−1^)	3.5 ± 0.19	4.1 ± 0.22
Specific growth rate (μ, h^−1^)	0.026 ± 0.03	0.024 ± 0.04
Cell yield (*Yx*/*s*, g g^−1^)	0.035 ± 0.02	0.041 ± 0.02
Ethanol yield (*Yp*/*s*, g g^−1^)	0.39 ± 0.37	0.43 ± 0.05
Volumetric substrate uptake (*Qs*, g l^−1^h^−1^)	6.25 ± 0.028	8.33 ± 0.07
Volumetric product productivity (*Qp*, g l^−1^h^−1^)	2.45 ± 0.06	3.6 ± 0.11
Specific sugar consumption rate (*q_*s*_*, g g^−1^ h^−1^)	2.17 ± 0.19	1.85 ± 0.09
Specific product formation rate (*q_*p*_*, g g^−1^ h^−1^)	0.848 ± 0.058	0.798 ± 0.06
Conversion rate (%) into ethanol	78.24 ± 0.57	86.5 ± 0.34

$$\begin{array}{lll} \mu & = & standardized\;value\;for\;specific\;growth\;rate\;of\;microorganism\\ Y_{X∕S} & = & \frac{Mass\;of\;biomass\;\left(yeast\;cell\right)\;formed}{Mass\;of\;substrate\;\left(glucose\right)\;consumed}\\ Y_{P∕S} & = & \frac{Mass\;of\;product\;\left(ethanol\right)\;formed}{Mass\;of\;substrate\;\left(glucose\right)\;consumed}\\ Q_{s} & = & Substrate\;\left(glucose\right)\;uptake\;\left(g\right)\;per\;liter\;of\;hydrolysate\;per\;hour\\ Q_{P} & = & Product\;formed\;\left(g\right)per\;liter\;of\;hydrolysate\;per\;hour\\ q_{s} & = & \frac{Mass\;of\;substrate\;\left(glucose\right)\;consumed}{Mass\;of\;biomass\;\left(yeast\;cell\right)\;formed}\;\mu \\ q_{p} & = & \frac{Mass\;of\;product\;\left(ethanol\right)\;formed}{Mass\;of\;biomass\;\left(yeast\;cell\right)\;formed}\;\mu \end{array}$$

Several researchers fermented various types of sugars for the production of ethanol using different isolated yeast strains. Krishnan et al. (1999) reported an ethanol concentration of 47.9 g l^−1^ with 0.46 g g^−1^ ethanol yield after 36 h of incubation period by using recombinant *Saccharomyces* 1400 (pLNH33), which are comparable to the present study. This shows the efficiency of the isolates *K. marxianus* NIRE-K1 and NIRE-K3, which was comparable to *Saccharomyces* 1400 (pLNH33). In this study, specific growth rate of yeast on glucose-xylose mixture was found to lie between the specific growth rate on glucose and specific growth rate on xylose. Similarly, Behera et al. (2014c) carried out anaerobic fermentation of both glucose and xylose sugar using newly isolated NIRE-GX1 yeast at 40°C temperature which showed 7.1 ± 0.6 g l^−1^ maximum ethanol concentration with complete utilization of glucose (20 g l^−1^) in 24 h of incubation period. However, the strain was capable of simultaneously using glucose and xylose in a mixture of glucose concentration of 14 g l^−1^ and xylose concentration of 6 g l^−1^, achieving maximum ethanol and xylitol concentration of 5.3 ± 0.5 g l^−1^ and 0.95 ± 0.32 g l^−1^, respectively in 72 h fermentation time. In the present study, both the isolates *K. marxianus* NIRE-K1 and NIRE-K3 are capable of growing and fermenting in the presence of both glucose and xylose. Also, Kumar et al. (2009b) isolated a yeast strain *Kluyveromyces* sp. IIPE453 (MTCC 5314) from soil samples collected from dumping sites of crushed sugarcane bagasse in Sugar Mill, showing growth and fermentation efficiency at high temperatures ranging from 45 to 50°C. In batch fermentation, the strain showed maximum ethanol concentration of 82 ± 0.5 g l^−1^ (10.4% v/v) on initial glucose concentration of 200 g l^−1^, and ethanol concentration of 1.75 ± 0.05 g l^−1^ as well as xylitol concentration of 11.5 ± 0.4 g l^−1^ on initial xylose concentration of 20 g l^−1^ at 50°C of temperature. This study showed the efficiency of the *Kluyveromyces* sp. IIPE453 (MTCC 5314) for the utilization of both the sugars for the production of ethanol and xylitol. The use of *K. marxianus* NIRE-K1 and NIRE-K3 in the present study have also signified in aerobic production of xylitol (data not shown). Tanimura et al. (2012) isolated a yeast strain ATY839, which showed 99.5% identity to that of *Candida shehatae* was capable of producing a substantial amount of ethanol with 71.6% yield at 37°C temperature using 2% glucose or xylose sugar. The use of both the isolates *K. marxianus* NIRE-K1 and NIRE-K3 may have profound effect on economics of the process due to their thermotolerant nature. In ethanol production, cooling costs have great effect, which makes the process expensive. Hence, by using these thermotolerant yeasts, cooling and distillation costs can be reduced during process development. Besides, higher saccharification and fermentation rates, continuous ethanol removal and reduced contamination have stimulated a search for routes to thermotolerant yeasts. Therefore, thermotolerant microorganisms that are able to ferment both glucose and xylose are required for efficient bioconversion of biomass to ethanol which could overcome to the limitations of well-known ethanologens such as *Saccharomyces cerevisiae* or *Zymomonas mobilis* due to their metabolic inefficiency.

## Conclusion

The screened and characterized thermotolerant isolates *K. marxianus* NIRE-K1 and NIRE-K3 has a great potential for bioethanol production in this context. The optimization of growth conditions w.r.t. temperature and pH using FCCD could increase ethanol yields to 0.39 and 0.43 g g^−1^ using *K. marxianus* NIRE-K1 and K3 yeast, respectively. The experimental results indicate that the pH and temperature exert significant effects on growth and bioethanol production yields. However, further studies on the physiology of the isolates using lignocellulosic hydrolysate, effect of fermentation inhibitors and metabolic flux analysis is required for the process development to exploit the potential of these isolates at commercial scale.

## Notations

**Table:** 

μ	Specific growth rate (h^−1^)
μ_max_	Maximum specific growth rate (h^−1^)
*S*	Rate limiting substrate concentration (g l^−1^)
*K_*s*_*	Saturation constant or half velocity constant or substrate utilization constant (g l^−1^)
*P*	Final ethanol concentration (g l^−1^)
*X*	Final biomass concentration (g l^−1^)
*Y_*x*∕*s*_*	Cell yield (g g^−1^)
*Y_*p*∕*s*_*	Ethanol yield (g g^−1^)
*Q_*s*_*	Volumetric substrate uptake (g l^−1^h^−1^)
*Q_*p*_*	Volumetric product productivity (g l^−1^h^−1^)
*q_*s*_*	Specific sugar consumption rate (g g^−1^ h^−1^)
*q_*p*_*	Specific product formation rate (g g^−1^ h^−1^)

### Conflict of interest statement

The authors declare that the research was conducted in the absence of any commercial or financial relationships that could be construed as a potential conflict of interest.
